# Forecasting Atrial Fibrillation: The Predictive Power of N-terminal Prohormone of Brain Natriuretic Peptide in a Five-Year Study

**DOI:** 10.7759/cureus.62515

**Published:** 2024-06-17

**Authors:** Fawad Akbar, Deepak Lal, Muhammad Arshad, Maryum Imran, Muhammad Haidar Zaman, Sauda Usmani, Moazama Shakeel Ahmed, Fahad R Khan

**Affiliations:** 1 Cardiology, Tehsil Headquarter (THQ) Tangi, Government of Khyber Pakhtunkhwa, Peshawar, PAK; 2 Cardiology, National Institute of Cardiovascular Heart Disease, Karachi, PAK; 3 Biochemistry, King Abdullah Teaching Hospital, Mansehra, PAK; 4 Health and Biological Sciences, Abasyn University, Peshawar, PAK; 5 Immunology, Nanjing Medical University, Nanjing, CHN; 6 Physiology, Pak Red Crescent Medical and Dental College, Lahore, PAK; 7 Internal Medicine, Mayo Hospital Lahore, Lahore, PAK; 8 Cardiology, Lady Reading Hospital, Peshawar, PAK

**Keywords:** nt-probnp and cardiac prognosis, early detection of atrial fibrillation, heart disease prevention, nt-probnp, atrial fibrillation prediction

## Abstract

Introduction

Atrial fibrillation (AF) is a major global health concern, and early prediction is essential for managing high-risk individuals. N-terminal prohormone of brain natriuretic peptide (NT-proBNP) has emerged as a crucial biomarker for predicting AF. While most studies have concentrated on cohorts already diagnosed with AF or other cardiac diseases, this research investigates the predictive value of NT-proBNP for AF development in a population without prior AF diagnosis.

Methods and materials

A five-year prospective observational study was conducted on 4090 individuals aged 45 to 75 with no previous diagnosis of AF. Baseline demographic characteristics, comorbid conditions, cardiac-specific measures, and NT-proBNP levels were systematically recorded. The primary endpoint was the onset of AF, confirmed through annual 12-lead ECG or 24-hour Holter monitoring. Univariate and multivariate analyses identified factors associated with AF onset.

Results

Out of the total population, 16.6% (679 individuals) developed AF. Notably, increased NT-proBNP levels (P=0.001), older age (P=0.001), and hypertension (P=0.001) were significantly associated with the onset of AF. The mean NT-proBNP levels in the AF group were significantly higher than in the non-AF group (P<0.001). The AF group also showed a higher mean age and a greater prevalence of hypertension (P<0.001 for both).

Conclusion

This study confirms the predictive value of NT-proBNP for AF onset in a non-AF population, highlighting older age and hypertension as significant risk factors for AF development. The findings underscore the potential of NT-proBNP not only as a predictive biomarker but also as a therapeutic target. These insights emphasize the potential role of NT-proBNP in early intervention and management strategies for AF, suggesting that future research should include additional variables, such as lifestyle factors and genetic predisposition, in assessing AF risk.

## Introduction

Atrial fibrillation (AF), a common cardiac rhythm disorder characterized by rapid and irregular heartbeats, significantly contributes to global morbidity and mortality [1]. Early diagnosis and prediction in high-risk individuals are essential for minimizing complications and enhancing patient outcomes [2].

Biomarkers have become effective diagnostic, prognostic, and therapeutic tools in several medical specialties, including cardiovascular disease. One such biomarker that has demonstrated significant benefit is the N-terminal pro-brain natriuretic peptide (NT-proBNP), a peptide hormone that ventricular myocardium cells produce in response to increased wall stress. In many conditions, including heart failure and myocardial infarction, NT-proBNP levels have been associated with cardiac function and prognosis [3].

Despite the extensive research on biomarkers, there are gaps in understanding their predictive value for AF, especially in populations without a prior AF diagnosis. Most studies have concentrated on cohorts already diagnosed with AF or other cardiac diseases [4]. This focus limits the generalizability of their findings to broader populations, leaving a critical gap in predicting AF in those without a prior diagnosis. Our study aims to address this gap by investigating the predictive value of NT-proBNP for AF development in a population without prior AF diagnosis, thereby underscoring our study's unique contribution.

Furthermore, while various biomarkers have been studied for their role in predicting AF, many fall short in terms of reliability and specificity. For instance, C-reactive protein (CRP) and other inflammatory markers have shown some predictive value but are often influenced by a range of non-cardiac factors, leading to potential inaccuracies [5]. In contrast, NT-proBNP has consistently demonstrated a stronger association with cardiac stress and AF onset, justifying our focus on this biomarker as a superior predictive tool [6].

This prospective observational research conducted over five years aimed to evaluate the predictive value of NT-proBNP levels for the onset of AF in a non-AF group. These results would clarify the relationship between NT-proBNP and the onset of AF, informing future AF prediction and prevention techniques in high-risk individuals.

## Materials and methods

To determine the predictive value of N-terminal pro-brain natriuretic peptide (NT-proBNP) concentration in relation to the development of atrial fibrillation (AF) in a group that initially exhibited no indications of AF, a longitudinal observation-based study was conducted from 2018 to 2023. We recorded data from 4090 individuals without a prior AF diagnosis, ranging in age from 45 to 75. The cohort consisted of 2040 males and 2050 females, approximately a 50/50 gender split. The age range of 45 to 75 years was selected due to the higher prevalence of AF in this demographic. AF incidence increases significantly with age, particularly after the age of 40, making this age group particularly relevant for studying the predictive value of NT-proBNP. Including individuals from 45 to 75 years ensures that the study captures a wide range of risk profiles while focusing on a population at substantial risk for developing AF.

Sample size

The chosen sample size of 4090 was determined based on power calculations to detect a clinically significant difference in NT-proBNP levels between individuals who develop AF and those who do not, with a power of 0.80 and an alpha of 0.05. This sample size ensures sufficient statistical power to identify meaningful associations while accounting for potential dropouts and non-compliance [7].

Ethical considerations

Ethical approval for the study was granted by the ethical review boards of all participating centers on June 1, 2018, and each participant provided informed consent. The study adhered to the principles outlined in the Declaration of Helsinki. Potential ethical issues included ensuring patient confidentiality, obtaining informed consent, and mitigating any psychological distress due to participation. Confidentiality was maintained by anonymizing data, and informed consent was obtained after thoroughly explaining the study's purpose and procedures. Any psychological distress was addressed by providing participants with the option to withdraw from the study at any time without any consequences.

Data collection

Demographic characteristics, such as age, gender, and body mass index (BMI), were collected at the start of the study from several setups to ensure a diverse representation. Blood pressure and heart rate were measured using an automated blood pressure monitor (Omron HEM-907XL, Omron Healthcare, Inc., Lake Forest, IL, USA) and a pulse oximeter (Nonin Onyx Vantage 9590, Nonin Medical, Inc., Plymouth, MN, USA). Standard laboratory procedures were conducted to test liver function (aspartate aminotransferase (AST) and alanine aminotransferase (ALT)), lipid status (cholesterol (CHOL), triglycerides (TG), low-density lipoprotein (LDL), high-density lipoprotein (HDL)), renal function (blood urea nitrogen (BUN) and creatinine (CRE)), and coagulation markers (international normalized ratio (INR), fibrinogen degradation products (FDP)). Blood samples were taken following an overnight fast, and NT-proBNP levels were measured using an electrochemiluminescence-based immunoassay (Roche Elecsys 2010, Roche Diagnostics, Indianapolis, IN, USA).

Participants’ medical histories were thoroughly examined with an emphasis on comorbid illnesses such as cerebral infarction, hypertension, diabetes, coronary heart disease, and renal disease. It was clarified that the study population did not have a prior diagnosis of AF or other heart failure conditions apart from coronary heart disease.

Two-dimensional echocardiography was utilized to collect cardiac-specific metrics, such as left atrial diameter, left ventricular diameter, ejection fraction (EF%), and cardiac output (CO), following the criteria set by the American Society of Echocardiography.

Data analysis

Independent t-tests were used for continuous data while chi-squared tests were employed for categorical variables. Continuous data were represented as mean and standard deviation (SD) while categorical variables were described as numbers (percentages). Univariate and multivariate analyses were performed for prediction. A p-value of 0.05 was considered statistically significant. All data analysis was conducted using the SPSS software package (version 26.0, IBM Corp, Armonk, NY, USA).

Surveillance and follow-up assessment

Every year, the onset of AF in the patients was confirmed using a 12-lead ECG or a 24-hour Holter monitor. The primary endpoint was the development of AF, with secondary outcomes including differences in comorbidity rates and cardiac-specific measures across groups. Participants were tracked for five years to examine the intervention’s long-term effects. Additionally, information on medication use and lifestyle variables like smoking and alcohol use were collected to assess their potential impact on the development of AF.

## Results

In this prospective longitudinal cohort study, conducted from 2018 to 2023, we evaluated 4090 individuals, aged 45 to 75, without a prior diagnosis of atrial fibrillation (AF) to assess the predictive value of NT-proBNP levels for the development of AF. The cohort included 2040 men and 2050 women, with an approximately equal gender distribution. The majority of the patients were seen on an outpatient basis, but a subset required short-term hospital admissions (mean duration of 2.5 days) for diagnostic purposes.

Out of the total population, 16.6% (679 individuals) developed AF over the five-year study period, with an average time to AF diagnosis of 2.8 years. The non-AF group consisted of 3411 subjects who did not develop AF. Baseline demographic and clinical characteristics indicated a modest male majority in both groups (55.7% in the AF group and 50.8% in the non-AF group). The AF group had a significantly higher mean age (66.57 ± 11.92 years) compared to the non-AF group (57.90 ± 13.07 years) (p < 0.001).

Table 1 shows the gender distribution, whereas Table 2 shows the age-wise distribution and physiological parameters of the AF and non-AF groups.

**Table 1:** Gender distribution by group AF: atrial fibrillation, N: count; %: percentage

Category	AF Group (N)	AF Group (%)	Non-AF Group (N)	Non-AF Group (%)
Total	679	-	3411	-
Gender (Males)	378	55.7%	1731	50.8%
Gender (Females)	301	44.3%	1680	49.2%

**Table 2:** Mean age and BMI by group AF: atrial fibrillation; BMI: body mass index; SD: standard deviation

Category	AF Group Mean (M)	AF Group Standard Deviation (SD)	Non-AF Group Mean (M)	Non-AF Group Standard Deviation (SD)
Age (years)	66.57	11.92	57.90	13.07
BMI (kg/m2)	25.08	4.72	25.09	3.32

Although heart rate and blood pressure were comparable, systolic blood pressure (SBP) was significantly greater in the non-AF group. The results of lab testing for liver enzymes (AST and ALT), lipid profiles, renal function tests, and coagulation markers differed somewhat between the two groups. Additional examination of the cohorts for comorbidities found that the prevalence of NT-ProBNP in the AF group (92.06%) was substantially greater than that in the non-AF group (33.93%). Additional comorbidities, such as cerebral infarction, hypertension, diabetes, coronary heart disease, and renal disease, had different prevalence rates in the two groups. Interestingly, hypertension was substantially more common in the non-AF group (p 0.05). Details are shown in Table 3.

**Table 3:** Prevalence of comorbid conditions and cardiac indices among the atrial fibrillation and non-atrial fibrillation cohorts AF: atrial fibrillation; NT-proBNP: N-terminal prohormone of brain natriuretic peptide

Category	AF Group Yes (N)	AF Group Yes (%)	AF Group No (N)	AF Group No (%)	Non-AF Group Yes (N)	Non-AF Group Yes (%)	Non-AF Group No (N)	Non-AF Group No (%)
NT-proBNP	626	92.06%	53	7.81%	1,158	33.93%	2,253	66.07%
Cerebral Infarction	50	7.4%	629	92.6%	48	1.4%	3,363	98.6%
Hypertension	351	51.7%	328	48.3%	2,750	80.7%	661	19.3%
Diabetes	120	17.7%	559	82.3%	484	14.2%	2,927	85.8%
Coronary Heart Disease	145	21.4%	534	78.6%	337	9.9%	3,074	90.1%
Kidney Disease	9	1.3%	670	98.7%	78	2.3%	3,333	97.7%

Cardiac metrics, such as left atrial diameter left ventricular diameter, ejection fraction (EF%), and cardiac output (CO) indicated minor differences, with the AF group having a considerably greater left atrial diameter.

Univariate analysis revealed that NT-proBNP levels in the AF group were significantly greater than in the non-AF group (P 0.001). Age (P = 0.001), hypertension (P = 0.002), and greater left atrial diameter (P = 0.001) were also strongly linked with the start of AF. Table 4 displays the detailed results.

**Table 4:** Univariate analysis of factors associated with AF onset AF: atrial fibrillation; NT-proBNP: N-terminal prohormone of brain natriuretic peptide; SD: standard deviation

Category	Condition	AF Group	Non-AF Group	P-value
Age (mean ± SD, years)		66.57 ± 11.92	57.90 ± 13.07	< 0.001
NT-proBNP (mean ± SD, pg/mL)		187.93 ± 71.47	67.59 ± 22.35	< 0.001
Hypertension	Yes	351 (51.7%)	2,750 (80.7%)	0.002
Hypertension	No	328 (48.3%)	661 (19.3%)	0.002
Left Atrial Diameter (mean ± SD, mm)		38.83 ± 6.34	32.22 ± 3.89	< 0.001

After correcting for other factors, increased NT-proBNP levels (hazard ratio (HR): 1.03, 95% CI: 1.01-1.05, P = 0.001), older age (HR: 1.05, 95% CI: 1.04-1.07, P = 0.001), and hypertension (HR: 1.26, 95% CI: 1.04-1.51, P = 0.02) were substantially linked with the development of AF. The findings are summarized in Table 5.

**Table 5:** Multivariable Cox regression analysis of factors associated with AF onset AF: atrial fibrillation; NT-proBNP: N-terminal prohormone of brain natriuretic peptide; HR: hazard ratio; CI: confidence interval

Category	HR (95% CI)	P-value
NT-proBNP (per 10 pg/mL increase)	1.03 (1.01-1.05)	0.001
Age (per year increase)	1.05 (1.04-1.07)	< 0.001
Hypertension (Yes)	1.26 (1.04-1.51)	0.02

## Discussion

The results of our study confirm the value of NT-proBNP as a significant biomarker for anticipating the onset of atrial fibrillation. High levels of NT-proBNP are significantly associated with an increased risk of AF development, particularly in people who initially showed no symptoms. The predictive value of NT-proBNP was further supported by the finding that those with elevated levels were more likely to experience AF sooner than those with normal levels. These results are consistent with earlier research [8-12].

Our study's multivariable Cox regression analysis also showed that hypertension and advanced age are still significantly correlated with the onset of AF. The results of earlier studies that identified older age and hypertension as independent risk factors for AF are confirmed by this conclusion [13,14]. Our study did not discover the same association as Polovina et al.'s findings, which identified diabetes mellitus as a significant predictor of AF [15].

Our study's AF and non-AF groups did not differ significantly in terms of cardiac measurements such as left atrial diameter, left ventricular diameter, ejection fraction, and cardiac output. The relatively healthy population we looked at, which had no known cases of AF or significant cardiac diseases, may be to blame for this discrepancy. However, Vaziri et al. (1994) and other earlier studies have demonstrated that a larger left atrial diameter is a significant predictor of AF [2,16-18].

Our study highlights the need for additional research into the contribution of other factors, such as lifestyle modifications, other comorbidities, and genetic predisposition, to the onset of AF [18-21]. Chua et al. suggested including these extra variables to enhance the prediction of AF risk [22]. We concur with this advice because relying solely on NT-proBNP levels could obscure AF's multifaceted, complex nature. The future development of a broader, more comprehensive risk assessment tool may result from this. Furthermore, it is crucial to determine whether our findings apply to younger demographics, given that most of our study participants were older. According to previous research, the ability of NT-proBNPs to predict AF may decline as people get younger [23-27]. Future research may therefore benefit from concentrating on younger populations or using a stratified approach to determine whether the NT-proBNP's predictive ability varies with age.

Last but not least, despite our study's statistically significant correlation between elevated NT-proBNP levels and the onset of AF, the precise pathophysiological mechanism underlying this association is still unknown. According to Patton and Ellinor et al., understanding these mechanisms may help create preventive measures or interventions that lessen the prevalence of AF [6]. Even as a potential therapeutic target, NT-proBNP merits further investigation.

Limitations

Despite our findings, several limitations should be noted. First, our study population consisted of relatively healthy individuals with no known cases of AF or significant cardiac diseases other than coronary heart disease, which may limit the generalizability of our results to broader populations. Additionally, although NT-proBNP is a strong predictor of AF, it is a marker of cardiac stress and not specific to AF, which means that elevated levels can also indicate other cardiac conditions, potentially leading to an overestimation of AF risk. Our study also did not include the left atrial volume index (LAVI), a recognized predictor of AF, due to equipment limitations and the need to maintain consistency across different study sites. Future studies should incorporate LAVI and other cardiac measurements to improve predictive accuracy. Finally, while adjustments for multiple comparisons were made using Bonferroni correction to minimize type I errors, the exact pathophysiological mechanisms linking elevated NT-proBNP levels to AF onset remain unclear and warrant further investigation.

Suggestions

Based on our findings, we recommend the routine measurement of NT-proBNP levels in patients aged 45-75 who are at high risk of AF, particularly those with hypertension and older age. NT-proBNP testing should be considered during hospital admissions, especially for patients presenting with symptoms suggestive of cardiac stress or those with significant comorbidities such as coronary heart disease. Additionally, NT-proBNP levels should be monitored during annual follow-ups or when new symptoms indicative of potential AF arise. This approach could facilitate early intervention and personalized management strategies, potentially reducing the incidence and complications associated with AF.

## Conclusions

This study confirms the predictive value of NT-proBNP for AF onset in a non-AF population, highlighting older age and hypertension as significant risk factors. The findings suggest that NT-proBNP can be used in screening programs to identify high-risk individuals, enabling early intervention and personalized management strategies. Integrating NT-proBNP testing into routine check-ups could improve AF prevention and management, emphasizing its clinical utility as a predictive biomarker. Our study adds to the growing body of research supporting NT-proBNP as a predictor of AF, particularly in individuals without a history of the condition. However, it is important to note that NT-proBNP is a marker of cardiac stress, not just AF. Various other cardiac conditions can present with elevated NT-proBNP levels, potentially overestimating AF risk. Thus, additional research is required to refine these findings and explore the potential of combining NT-proBNP with other biomarkers for more precise AF prediction.
